# Neurophysiological Response of Adults with Cerebral Palsy during Inclusive Dance with Wheelchair

**DOI:** 10.3390/biology11111546

**Published:** 2022-10-22

**Authors:** Sandra Mendoza-Sánchez, Alvaro Murillo-Garcia, Juan Luis Leon-Llamas, Jesús Sánchez-Gómez, Narcis Gusi, Santos Villafaina

**Affiliations:** 1 ASPACE Badajoz (Asociación de Personas con Parálisis Cerebral), 06011 Badajoz, Spain; 2 Grupo de Investigación Actividad Física y Calidad de Vida (AFYCAV), Facultad de Ciencias del Deporte, Universidad de Extremadura, 10003 Cáceres, Spain; 3 Departamento de Desporto e Saúde, Escola de Saúde e Desenvolvimento Humano, Universidade de Évora, 7004-516 Évora, Portugal

**Keywords:** EEG, stimulation, disability, music, dance

## Abstract

**Simple Summary:**

Electroencephalography (EEG) has been considered a useful methodology for the evaluation of cerebral palsy therapeutic strategies. Cerebral palsy is a neurodevelopment disorder caused by a nonprogressive damage to the developing brain. In this sense, the aim of the present article is to study the EEG response of people with cerebral palsy during an inclusive dance with a wheelchair as well as to compare EEG power spectra between baseline, inclusive dances and listening to music. A total of 16 adults with cerebral palsy participated in three conditions: baseline, listening to music and performing an inclusive dance choreography with wheelchair. Results showed higher brain electrical activity at theta, alpha, and beta bands in dance conditions when compared to a baseline.

**Abstract:**

A total of 16 adults with cerebral palsy (age = 37.50 (7.78)) participated in this cross-sectional study. The electroencephalographic (EEG) data were recorded under three conditions: (1) baseline; (2) while listening to music; (3) while performing inclusive dance choreography with wheelchair. EEG data was banded into theta (4–7 Hz), alpha (8–12 Hz), and beta (13–30 Hz). Significantly higher values of theta, alpha, and beta bands were found in dance conditions than in the baseline. Significant differences between baseline and listening to music conditions were not found in any of the power spectrum bands. Differences between listening to music conditions and inclusive dance with wheelchair were observed in theta and beta power spectrum band studies in the F4 electrode. Inclusive dance with wheelchair increases theta, alpha, and beta power spectra when compared to baseline. In addition, the beta power spectrum is greater only during inclusive dance conditions, which could be modulated by emotions. However, future studies should corroborate this hypothesis.

## 1. Introduction

Cerebral palsy (CP) is a neurodevelopment disorder caused by nonprogressive damage to the developing brain which occurs prenatally, perinatally, or postnatally [1]. CP can lead to different problems, such as motor or intellectual impairments [2,3,4]. In order to reach the maximum potential as well as to prevent functional deterioration, different therapies have been provided [5]. 

Physical activity, hydrotherapy, constraint-induced movement therapy, bimanual therapy, and sensory stimulation have shown to be effective on CP [6,7,8]. However, due to the heterogeneity of the injury, is unlikely that a single rehabilitation fits all CP patients. Thus, new potential rehabilitation techniques have been encouraged. Electroencephalography (EEG) is a rapid and highly feasible technique. EEG is considered a useful methodology for the evaluation of CP therapeutic strategies [9]. 

Previous studies have investigated the role of dance interventions [10,11] and music in patients with CP [12,13]. In addition, dance increased proprioception, balance, posture, sensorimotor function, working memory, and aesthetic expression among other factors [14]. However, people with CP present difficulty in being engaged in top-down approaches, and a bottom-up approach is necessary. Therefore, the effects of wheelchair dance on CP have been poorly described. In this regard, a previous study focused on aerobic capacity after an intervention with wheelchair dance showed an improvement in aerobic capacity [15]. Moreover, other studies have been focused on wheelchair dance as an educational tool [16,17]. 

Therefore, this study aimed to study the EEG response of people with CP during inclusive dance with a wheelchair as well as to compare EEG power spectra between listening to music and inclusive dance with a wheelchair. Results would help to characterize an alternative therapy involving movement and musical stimulus for people with CP.

## 2. Materials and Methods

### 2.1. Participants

Our primary outcomes are the EEG power spectra (theta, alpha, and beta bands). In this regard, the vast majority of the studies which analyze EEG power spectrum used topographic images to report the results. Therefore, quantitative data that can serve as input to sample size calculators (such as G*Power) are not available. In addition, there are not previous studies which analyzed the EEG response to inclusive dance in adults or children with CP. All these reasons together make it impossible to perform an adequate sample size calculation based on previous studies in the field. Thus, we decided to include a convenient sample of people with CP that fulfilled the inclusion criteria and gave written informed consent to participate in this cross-sectional study until January 2020.

Therefore, a total of 16 adults (11 females and 5 males) with CP participated in this cross-sectional study. The mean age of the participants was 37.50 (7.78). The following inclusion criteria were set: (1) people with CP; (2) mild to severe cognitive impairment; and (3) low motor function. Exclusion criteria were: (1) low cognitive impairment; (2) autonomy; and (3) not suffering from CP. 

Procedures were approved by the University of Extremadura research ethics committee and parents/legal guardian gave written informed consent to participate in the study (approval number: 116/2018). Characteristics of participants are described in Table 1.

### 2.2. Procedure

EEG was recorded under three conditions:

(1) At baseline for two minutes with open eyes.
(2) While performing inclusive dance choreography with an occupational therapist and a kinesiologist. Under this condition, the specialist was next to the chair, in parallel and facing the front, so that constant visual contact was established with the patients. The specialists moved the chair, accompanying them in a dance that includes linear movements and turns. In addition, a ribbon tied to the chair was included for certain moments of the dance in which the professionals moved away from the chairs and later pulled it to continue with spins.
(3) While listening to music for two minutes. The selected songs in both conditions (under choreography and listening to music conditions) were not the same in order to avoid mental associations with the choreography. Nevertheless, a song of the same artist was selected to maintain the tone and style (114 beats per minutes for the choreographed song and 104 beats per minutes for the music condition). The order of inclusive dance and listening to music was randomized. All participants underwent the same choreography and listened to the same music.

### 2.3. Tests and Instruments 

CP severity was classified according to the gross motor function classification system (GMFCS) [18]. The GMFCS classifies movement into five levels: level I (walks without restrictions), level II (walks without restrictions but with limitation outdoors), level III (walks with assistive mobility devices), level IV (self-mobility with limitations), and level V (self-mobility is severely limited even using assistive devices).

The gross motor function measure-88 (GMFM-88) is an 88-item scale which evaluates the gross motor function in people with CP [19]. It comprises five dimensions: lying and rolling, sitting, crawling and kneeling, standing and walking, and running and jumping. The higher the score, the higher the gross motor function. 

The short sensory profile (SSP), a shortened form of Dunn’s sensory profile questionnaire [20], was used to screen sensory processing difficulties [21]. The SSP questionnaire contains 38 items organized into seven subscales; the greater score, the lower the sensory processing difficulty. The SSP total score was calculated for each participant.

The pediatric evaluation of disability inventory (PEDI) [22] was used to evaluate self-care, mobility, and social function. The PEDI questionnaire includes three domains (self-care, mobility, and social function) and consists of 197 items. Each item is dichotomous (1: capable and 0: incapable). 

The Enobio^®^ EEG system (Neuroelectric, Barcelona, Spain) with the Neuroelectrics^®^ instrument controller (NIC1) (Neuroelectrics, Barcelona, Spain) software was employed to monitor the EEG. Nineteen scalp locations, according to the International 10–20 system, were recorded using dry electrodes [23]. The electrodes were placed in frontal (Fz, F3, F4, F7, and F8), central (Cz, C3, and C4), temporal (T3, T4, T5, and T6), parietal (Pz, P3, and P4), and occipital (O1 and O2) areas. 

Two reference electrodes placed in the mastoids served as reference. A 250 Hz sampling rate was used. For pre-processing and data analysis, the EEGlab toolbox (MatLab) [24] was used. A 1 Hz high-pass filter was applied, and the cleanline algorithm in EEGlab was used to remove line noise. Artifact subspace reconstruction (ASR) was used to reject bad channels and correct continuous data, the bad channels were interpolated and data were re-referenced to average. The single equivalent current dipoles were estimated, the symmetrically constrained bilateral dipoles were searched, and the adaptive mixture independent component analysis (AMICA) was employed [25]. The IClabel algorithm was used to classify independent components (ICs) into seven categories (brain, muscle, eye, heart, line noise, channel noise, and other) [26]. The ICs were removed if dipoles were located outside the brain or were classified as muscle, heart, eye, channel noise, line noise, or another artifact. In addition, ICs were removed if the dipoles’ residual variance was larger than 15%. The power spectral densities were computed and banded into three different frequency bands: theta (4–7 Hz), alpha (8–12 Hz), and beta (13–30 Hz).

### 2.4. Statistical Analysis

To analyze the EEG data, the EEGlab toolbox (MatLab) was used [24]. Due to the difficulty of assuming the Gaussian distribution in the EEG data and the recommendation of Kitchen [27] of using non-parametric tests in biomedical research, non-parametric analyses (permutation analysis) were performed. In addition, to control type 1 error, false discovery rate correction (FDR), based on the Benjamini–Hochberg procedure, was performed. Pairwise comparisons between the three conditions (baseline, inclusive dance, and listening to music) were conducted to explore neurophysiological differences. In addition, in order to explore the homogeneity of the sample, participants were divided into two groups according to GMFM level (lower or higher than three). Comparisons between these two groups in the three conditions are reported as Supplementary Materials.

## 3. Results

Table 1 shows the participants’ characteristics. The results of the GMFM evidenced that participants have high motor restriction, and according to the PEDI, the participants showed low functionality. Regarding sensory impairments, all the participants ranged between “typical” and “probably impaired” according to the SSP questionnaire, so dramatic sensory impairment was not generally observed.

Differences between people who had lower GMFM levels (lower than three) and participants who had higher GMFM levels (higher than three) were not observed at baseline for theta, alpha, or beta power spectra (see Supplementary Figure S1). In addition, differences between these two groups while listening to music or during the inclusive dance were not found (see Supplementary Figures S2 and S3).

**Figure 1:**
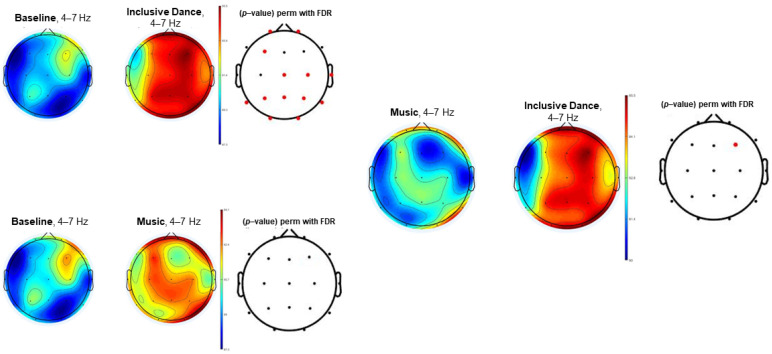
EEG topographic maps in the theta power spectrum (4–7 Hz) of the comparison between baseline, inclusive dance, and listening to music conditions. Significant comparisons (*p*-value < 0.05) have been highlighted in red.

**Figure 2:**
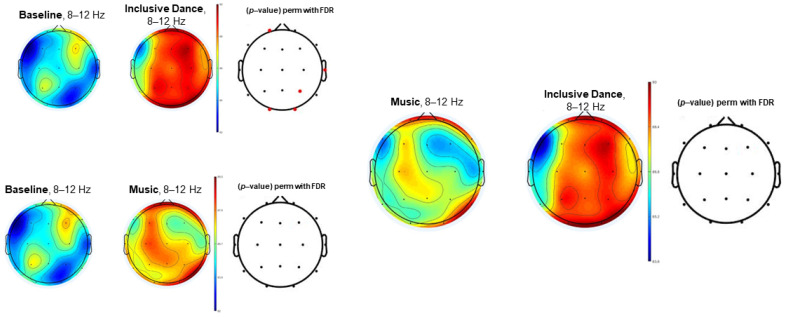
EEG topographic maps in the alpha power spectrum (8–12 Hz) of the comparison between baseline, inclusive dance, and listening to music conditions. Significant comparisons (*p*-value < 0.05) have been highlighted in red.

**Figure 3:**
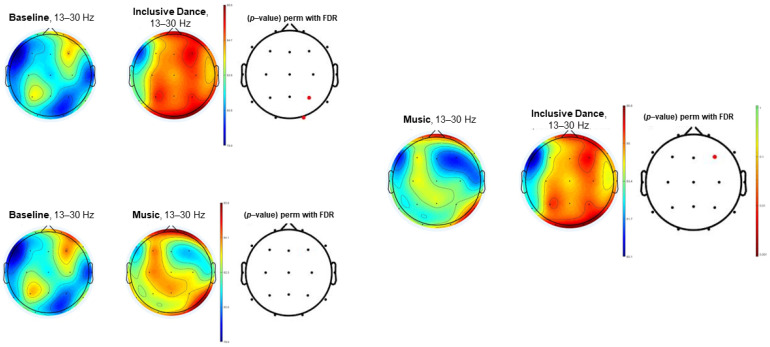
EEG topographic maps in the beta power spectrum (13–30 Hz) of the comparison between baseline, inclusive dance, and listening to music conditions. Significant comparisons (*p*-value < 0.05) have been highlighted in red.

Figure 1, Figure 2 and Figure 3 (theta, alpha, and beta, respectively) show the EEG topographic maps of the baseline, music, and inclusive dance conditions.

Figure 1 shows the topographic map in the EEG theta power spectrum (4–7 Hz) of the comparison between baseline and both inclusive dance and listening to music as well as the comparison between these two conditions (dance vs. music). Inclusive dance conditions showed a general higher theta EEG power spectrum in Fp1, Fp2, F3, Cz, C4, T4, Pz, P4, P3, T6, T5, O1, and O2 electrodes when compared to baseline conditions (*p*-value < 0.05). In the same line, music conditions showed higher theta EEG power spectrum results than baseline conditions. However, the significance level was not reached in any of the electrodes. In addition, higher activation can be observed in the dance conditions than in the music conditions, achieving significant differences in the F4 electrode (*p*-value < 0.05).

Figure 2 shows EEG topographic maps in the alpha power spectrum (8–12 Hz) of the comparison between baseline, inclusive dance, and listening to music conditions. Inclusive dance conditions showed significantly higher EEG alpha power spectrum results than baseline (*p*-value < 0.05) in the Fp1, T4, P4, O1, and O2 electrodes. Listening to music exhibited greater alpha power spectrum results than baseline, although differences did not reach the significance level (*p*-value > 0.05). Differences were not found between dance and music conditions in the alpha EEG power spectrum (*p*-value > 0.05).

Figure 3 shows EEG topographic maps in the beta power spectrum (13–30 Hz) of the comparison between baseline, inclusive dance and listening to music conditions for the beta EEG power spectrum. Significant differences (*p*-value < 0.05) were found between baseline and inclusive dance conditions in the P4 and O2 scalp locations. Significant differences were not observed in beta EEG power spectrum results between baseline and listening to music conditions. Significant difference between listening to music and inclusive dance conditions was observed in the F4 electrode (*p*-value < 0.05).

## 4. Discussion

The aim of this study was to compare the EEG power spectrum of people with CP during both listening to music and inclusive dance with wheelchair. Results showed that EEG power spectrum results in the theta, alpha, and beta power spectrum bands are greater in inclusive dance conditions than in the baseline conditions. Significant differences between listening to music and baseline conditions were not observed. In addition, differences between listening to music and inclusive dance with wheelchair were observed in theta and beta power spectrum bands in the F4 electrode.

Dance interventions have been previously used in the CP population to improve relevant motor aspects, such as balance, posture, proprioception, and motor timing [14]. Moreover, dance has been included in physical and occupational therapies due to the benefits in enjoyment and participation—and, therefore, motivation [10,11,28]. However, we are not aware of studies on the impact of inclusive dance with wheelchair on the EEG power spectrum of patients with CP. Our study showed that during inclusive dance, EEG power spectrum results at theta, beta, and alpha, were greater than during the baseline. In this regard, previous studies showed abnormalities between healthy controls and children with CP in the EEG power spectrum [29,30,31]. Regarding the alpha power spectrum, a previous study showed lower alpha and theta power spectra in children with CP than healthy controls [29]. This is relevant because alpha band has been considered to reflect cortical function [32]. Moreover, beta band has been associated with emotional tasks and stimuli [33,34]. In this regard, in both healthy controls and people with depression, increases in the beta power spectrum located in posterior brain areas were found during the processing of happiness emotion [35]. This is quite relevant because our results showed that beta power spectrum results significantly increased during inclusive dance in the right parietal and occipital brain areas (P4 and O2), whereas during music stimulation, these differences were not found. This could mean that inclusive dance could elicit positive emotion in patients with CP.

In this line, one of the most relevant findings of the present study is that participants achieved greater brain activity, which could be related to positive emotions during the inclusive wheelchair dance. However, the included participants, due to CP injuries, could not be totally involved in learning the choreography or following the rhythm of the music. The body movements performed by patients were free and may be induced by the emotion generated by dance. However, many studies have been focused on the physical benefits of dance on CP (due to motor impairments, such as balance, postural control, aerobic fitness, and gait performance) [15,36,37,38,39]. Thus, the present study can generate a line of investigation based on the benefits of dance on brain activity related to the emotionality of inclusive dance regardless of the motor complexity of the practice. 

Auditory stimulation and music have been used as a therapy for improving motor function in CP [12,13]. Moreover, music could have a significant impact on brain oscillations. Condition in which participants were listening to music were included in order to take into account this possible confounder. In this regard, significant differences between music and baseline conditions did not reach the significance level in any of the frequency bands studies, albeit activations seemed to be greater during inclusive dance. However, differences between listening to music and inclusive dance with a wheelchair were observed in theta and beta power spectrum bands in the F4 electrode. These results are quite relevant since a recent systematic review stated that frontal and parietal areas seem to store the most information about emotional states, highlighting the role of beta power spectrum [40]. Moreover, Ameera, et al. [41] used the F4 power spectrum to discriminate between pleasure and displeasure states, obtaining 97.5% of accuracy in the beta waves (in electrode F4) in discriminating between these states (higher values of the beta power spectrum were found in the pleasure state). In this line, future research should study the role of the beta power spectrum and emotion induced by dance.

This study presents some limitations which should be considered. Firstly, the characteristics of CP make it almost impossible to achieve a homogeneous sample. However, the sub-group analyses reported in the Supplementary Materials did not show significant differences between people with lower and higher GMFCS levels in brain electrical patterns. In addition, only the greater differences reached the significance level due to the FDR procedure for multiple comparisons and the relatively small sample size.

## 5. Conclusions

Inclusive dance with a wheelchair and listening to music increase theta, alpha, and beta power spectra in adults with CP when compared to baseline. Significant differences were not observed between listening to music and baseline, albeit activations seemed to be greater under listening to music conditions. Differences between listening to music and inclusive dance with a wheelchair were observed in theta and beta power spectra in the F4 electrode. These differences could be explained by emotional processes induced by dance. 

## Figures and Tables

**Table 1:** Characteristics of participants.

Variable	Mean (SD)
Age (years)	37.50 (7.78)
PEDI	69.68 (51.01)
SSP	148.87 (7.69)
GMFM-88	39.19 (25.96)
GMFCS levels	N (Percentage)
Level I	0 (0%)
Level II	9 (56.25%)
Level III	0 (0%)
Level IV	3 (18.75%)
Level V	4 (25%)
Sex, N (%)	Females: 11 (68.75%)
	Males: 5 (31.25%)

GMFM: gross motor function measure; GMFCS: gross motor function classification system; SSP: short sensory profile; PEDI: pediatric evaluation of disability inventory; N: number of participants.

## Data Availability

Not applicable.

## Supplementary Materials

The following supporting information can be downloaded at: https://www.mdpi.com/article/10.3390/biology11111546/s1, Figure S1: EEG topographic maps of participants with low and higher GMFCS level in the theta (4–7 Hz), alpha (8–12), and beta (13–30) power spectra at baseline; Figure S2: EEG topographic maps of participants with low and higher GMFCS level in the theta (4–7 Hz), alpha (8–12), and beta (13–30) power spectra while listening to music; Figure S3: EEG topographic maps of participants with low and higher GMFCS level in the theta (4–7 Hz), alpha (8–12), and beta (13–30) power spectra during inclusive dance.
