# Supplementary material for: Neurophysiological Response of Adults with Cerebral Palsy during Inclusive Dance with Wheelchair
Source: Biology (Basel). 2022 Oct 22;11(11):1546. doi: 10.3390/biology11111546 (PMC9687850; doi:10.3390/biology11111546)
Supplement: Supplementary file 1 [file biology-11-01546-s001.zip › biology-1947337-supplementary.pdf]

Supplementary Material

Figure S1 shows the comparison between participants with lower and higher level of GMFCS at baseline. Significant differences were not found.

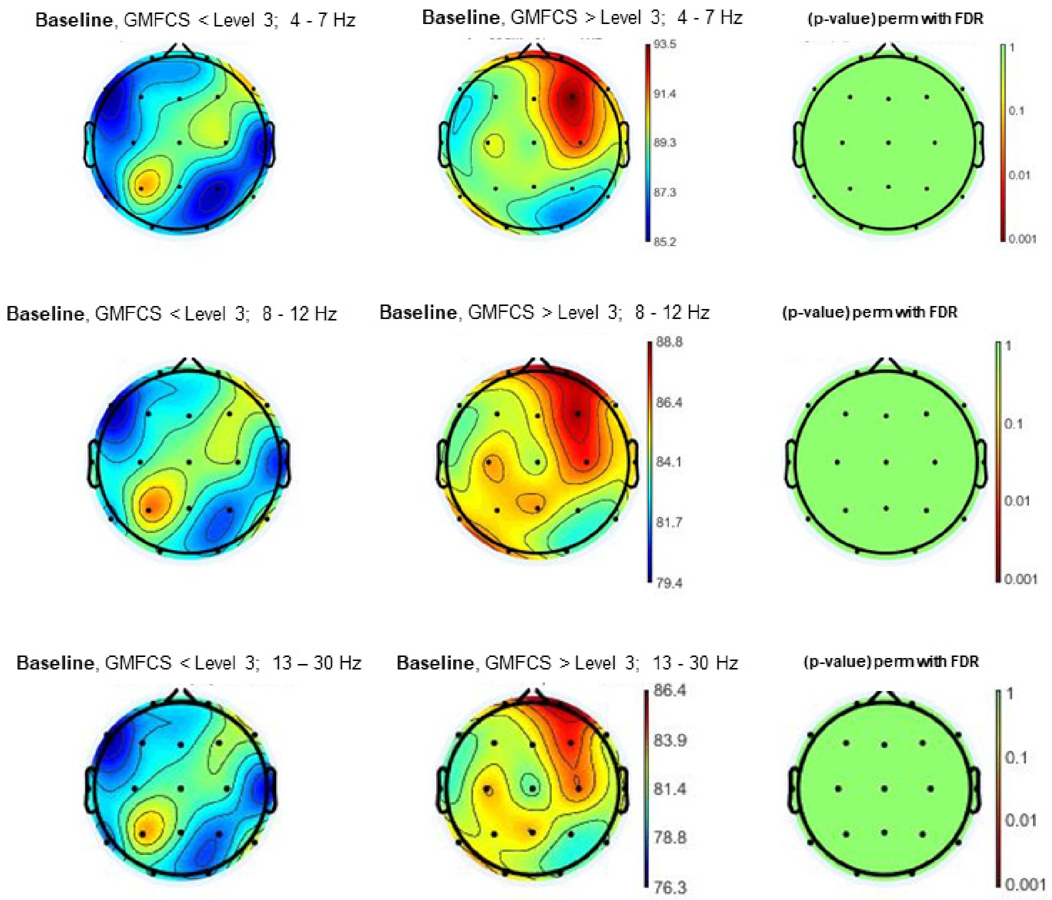

Figure S1. EEG topographic maps of participants with low and higher GMFCS level in the theta (4-7 Hz), alpha (8-12) and beta (13-30) power spectrums at baseline.

Figure S2 shows the comparison between participants with lower and higher level of GMFCS while listening to music. Significant differences were not found in any of the power spectrum bands included in the present article.

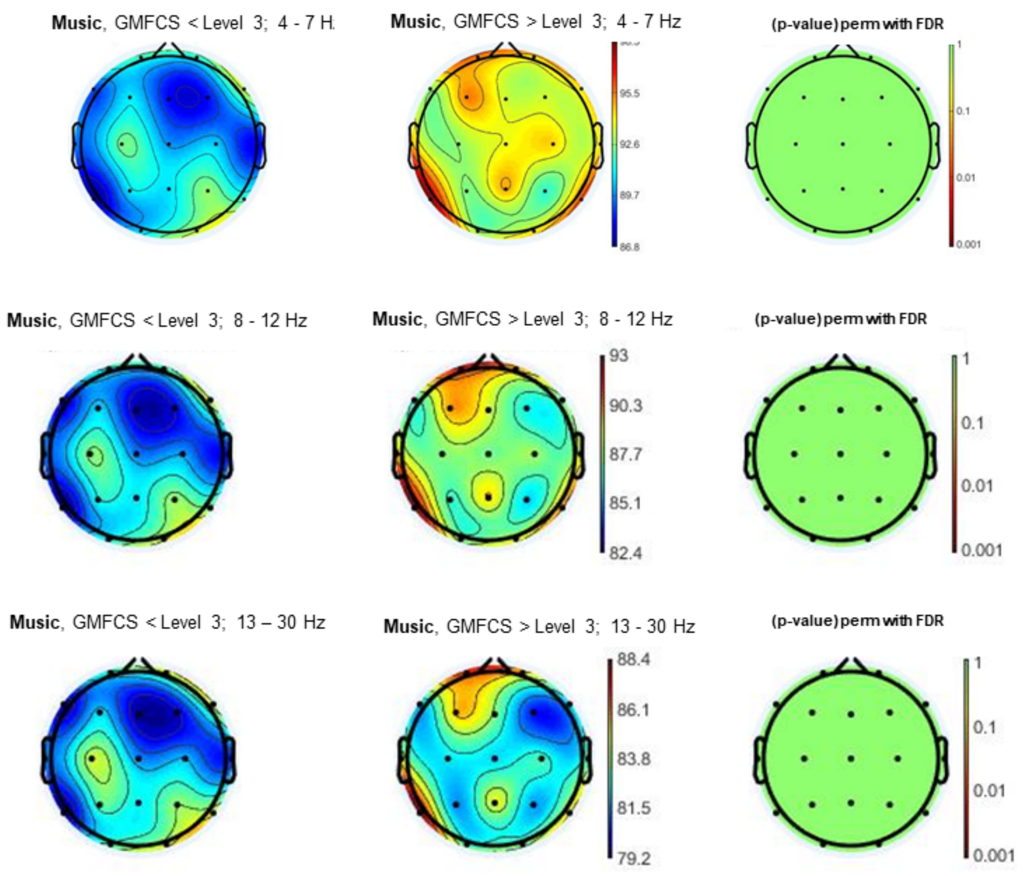

Figure S2. EEG topographic maps of participants with low and higher GMFCS level in the theta (4-7 Hz), alpha (8-12) and beta (13-30) power spectrums while listening to music.

Figure S3 shows the comparison between participants with lower and higher level of GMFCS during inclusive dance. Significant differences were not found.

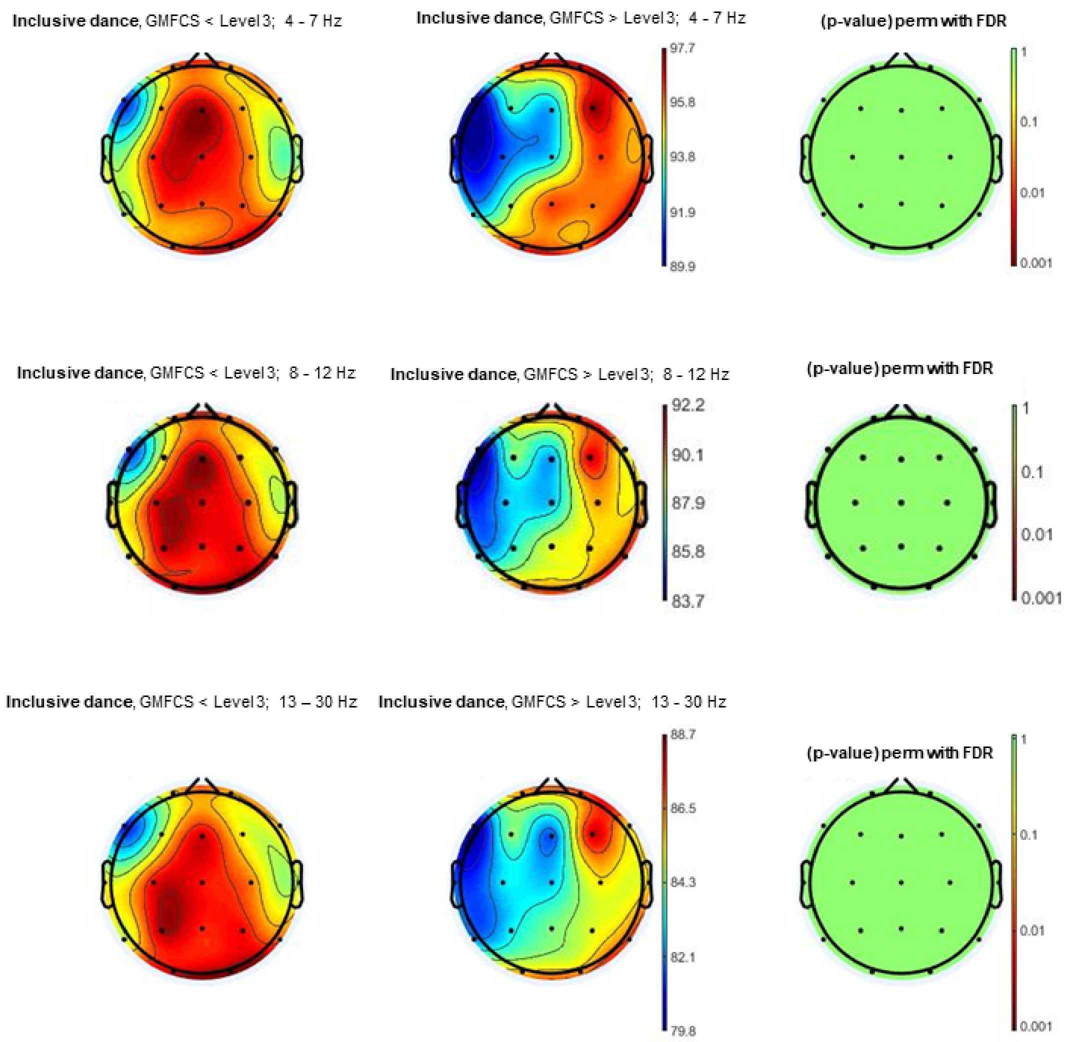

Figure S3. EEG topographic maps of participants with low and higher GMFCS level in the theta (4-7 Hz), alpha (8-12) and beta (13-30) power spectrums during inclusive dance.
